# Exploring cadmium exposure as a contributor to male infertility: insights from the relationship between environmental pollutants and the male reproductive system

**DOI:** 10.3389/fendo.2026.1819705

**Published:** 2026-07-22

**Authors:** Chenming Zhang, Jing Hu, Sicheng Ma, Yifei Wang, Heng Liu, Yinuo Zhang, Wenlin Yu, Yizhe Gao, Jun Lu

**Affiliations:** 1 Henan University of Chinese Medicine, Zhengzhou, Henan, China; 2 Auckland Bioengineering Institute, University of Auckland, Auckland, New Zealand

**Keywords:** blood-testis barrier (BTB), cadmium, environmental exposure, human equivalent dose (HED), male infertility, oxidative stress

## Abstract

This paper is a narrative review focusing on the association between environmental cadmium exposure and male reproductive dysfunction. The relationship between environmental pollutants encountered in daily life and the male reproductive system is attracting increasing attention. Humans are exposed to cadmium through inhalation, dietary intake (food) and other routes, leading to adverse health effects. This study systematically searched PubMed, Web of Science, and CNKI databases from January 1, 2020, to December 31, 2024, and included 40 eligible *in vivo* studies based on standardized selection criteria. This study reviewed and analyzed relevant animal experiments conducted in recent years. Based on the lowest observable adverse effect level (LOAEL, 10 μg/kg/d) for reproductive toxicity in rats from a chronic 90−day study (Ait Benbella et al., 2025), the minimum critical human equivalent dose (HED) of cadmium−induced male reproductive damage was calculated, All 40 studies in **Table 1** were selected according to predefined inclusion/exclusion criteria (see Section 2). The Lowest-Observed-Adverse-Effect Level (LOAEL) was converted to HED using the following formula: $HED(mg/kg)=Animal\;LOAEL\;(mg/kg)\times \frac{km\;of\;animal\;}{km\;of\;human}$ (The LOAEL was derived from rat reproductive toxicity data (10 μg/kg/d) (Ait Benbella et al., 2025); km factors of 6 (rat) and 37 (human, 60kg) were applied. The calculated HED was 0.002 mg/kg/d.) Comparisons reveal that cadmium exposure levels in some regions approach or even exceed this threshold. The results suggest that cadmium may cause damage to the male reproductive system, potentially adversely affecting semen quality. Cadmium also induces renal dysfunction that indirectly exacerbates testicular injury via the renal−testis axis, further disrupting the hypothalamic−pituitary−testicular axis and amplifying reproductive toxicity. These findings highlight the integrated renal−reproductive toxicity of environmental cadmium exposure.

## 1 Introduction

In contemporary society, male infertility has become a significant social concern. Male infertility, a multifactorial disease, affects approximately 7% of the male population (1). Environmental pollutants may disrupt male reproductive function, potentially reducing sperm quality, impairing testicular structure, and increasing sperm DNA fragmentation (2). As a widespread environmental toxicant, cadmium exerts reproductive toxicity through multiple interconnected pathways in experimental models, including oxidative stress, spermatogenic cell apoptosis, testicular inflammation, androgen suppression, ion homeostasis imbalance, epigenetic dysregulation, vascular endothelial damage, and blood-testis barrier disruption, and indirect amplification of testicular injury via the renal−testis axis (3).

Cadmium (Cd) is a toxic and hazardous heavy metal element, widely present in the natural environment and daily life. In the natural environment, Cd primarily originates from sources such as volcanic eruptions and rock weathering (4). In recent years, with the development of urban industrialization, human activities including mining, smelting, and the refining of zinc-containing ores have led to a significant increase in Cd emissions into the environment (5). Furthermore, Cd exhibits high solubility in soil, making it readily absorbed by plants like rice, and subsequently accumulated in the human body through the food chain (6).

Heavy metals represent a significant toxicological factor affecting fertility. Bellas et al. indicated that approximately 5% of infertility cases are associated with exposure to harmful effects such as ultra-high-frequency radiation, heavy metals, as well as herbicides and pesticides on farms (7). Certain occupations, such as those in radiology, industrial and chemical solvent plants, refineries, and lead mines, may also interfere with an individual’s fertility status (8, 9). Air pollutants, including exhaust fumes and smog from vehicles, can cause defects in gametes in both males and females (10). Population analyses in China reveal that adults and adolescents aged 14 and above are more vulnerable, with males exhibiting higher cadmium concentrations (11). Humans are exposed to metal aerosols, including lead (Pb^2+^) and cadmium (Cd^2+^), both occupationally and environmentally (12). These toxins accumulate in the male reproductive organs. Globally, the toxicity associated with cadmium and its negative impacts on humans and animals cannot be underestimated. Heavy metals including cadmium, chromium, and lead exert their toxicity mainly through inducing oxidative stress, which disrupts the antioxidant system and causes tissue damage. Selenium has been confirmed to serve an important antioxidative role against heavy metal-induced toxicity (13).

This review aims to systematically integrate multidisciplinary evidence regarding cadmium (Cd)-induced male infertility. By critically evaluating population studies and experimental models, it seeks to establish a dose-effect relationship between Cd exposure and impairment of sperm parameters, and to address controversial issues such as the effects of low-dose chronic exposure and genetic susceptibility factors. Secondly, it will elucidate the cascade mechanisms underlying Cd’s reproductive toxicity, encompassing oxidative stress-driven apoptosis of spermatogenic cells, disruption of the blood-testis barrier, dysregulation of epigenetic mechanisms, and interference with ion channels. Finally, the review will identify research gaps and outline future directions.

## 2 Literature search and study selection criteria

A systematic literature search was performed in PubMed, Web of Science, and CNKI databases from January 1, 2020, to December 31, 2024. Search terms included “cadmium,” “male reproductive toxicity,” “testis,” “sperm,” “rodent,” “rat,” “mouse,” “oral exposure,” and “dose−response”.

### 2.1 Inclusion criteria

1. *In vivo* studies using sexually mature male rats or mice.
2. Oral cadmium exposure (drinking water or diet) consistent with environmental exposure.
3. Reported at least one reproductive or renal endpoint: sperm quality, testicular injury, blood-testis barrier disruption, or renal dysfunction.
4. Clear quantitative exposure dose and outcome data available.

### 2.2 Exclusion criteria

1. *In vitro* or cell−line studies.
2. Non−oral exposure (intraperitoneal, subcutaneous, or intravenous injection only).
3. No explicit dose or sample size information.
4. Studies focusing solely on non−reproductive endpoints.
5. Duplicate publications or overlapping animal cohorts.
6. A total of 40 studies meeting all criteria were included in **Table 1**.

**Table 1:** Cadmium exposure doses in male rodents and corresponding human equivalent doses (HEDs ) (in chronological order).

Number	Literature	Animal	Dosage	Exposure days	Effect(s)	HED(Results are rounded to three decimal places)
1	Evaluation of the Reversibility of Cadmium-Induced Testicular Toxicity Following Recovery Alone or with Zinc Supplementatio (99)	rats	10 μg/kg/d	90	ABDEF^1^	0.002mg/kg/d
2	Early Zinc Supplementation Enhances Epididymal Sperm Glycosylation, Endocrine Activity, and Antioxidant Activity in Rats Exposed to Cadmium (100)	rats	0.5mg/kg	22	EG	0.081mg/kg/d
3	Protective Effect of Lycium Barbarum Polysaccharides and Sodium Selenite on Cadmium Chloride-Induced Testicular Toxicity in Rat (101)	rats	0.5mg/kg	7-84	FGH	0.081mg/kg/d
4	Modulation of AMPK/mTOR Autophagic Pathway Using Dapagliflozin Protects Against Cadmium-Induced Testicular and Renal Injury in Rats (102)	rats	30 mg/kg	21	FJ	4.839mg/kg/d
5	Targeting TLR4/NF-κB signaling, oxidative stress, and apoptosis by farnesol mitigates cadmium-induced testicular toxicity in rats (103)	rats	1.2 mg/kg	1	FGH	0.194mg/kg/d
6	The salutary action of vitamin E on reproductive performance and renal functions in cadmium-exposed male mice (104)	mice	3.5 mg/kg	60	ABEFGJ	0.285mg/kg/d
7	Effect of Melatonin and Ginseng on rat testis and sperm quality against cadmium toxicity via inhibiting oxidative stress and autophagy pathways (105)	rats	2.0 mg/kg	1	ABCH^2^	0.323mg/kg/d
8	Mitigative effects of didymin against cadmium-induced renal injury via regulating Nrf-2/Keap-1, apoptosis, inflammation and oxidative stress (106)	rats	5 mg/kg	30	HJ	0.806mg/kg/d
9	Astragaloside IV attenuates cadmium induced nephrotoxicity in rats by activating Nrf2 (107)	rats	5 mg/kg	14	HJ	0.806mg/kg/d
10	Effect of propolis supplementation on cadmium toxicity associated with renal and hepatic dysfunction in rats (108)	rats	1.76 mg/kg	28	GJ	0.284mg/kg/d
11	Anacyclus pyrethrum enhances fertility in cadmium-intoxicated male rats by improving sperm functions (109)	rats	1mg/kg	56	DGH	0.161mg/kg/d
12	Diallyl disulfide prevents cadmium-induced testicular injury by attenuating oxidative stress, apoptosis, and TLR-4/NF-κB and JAK1/STAT3 signaling and upregulating SIRT1 in rats (110)	rats	1.2 mg/kg	14	GH	0.194mg/kg/d
13	Nanostructured biloalbuminosomes loaded with berberine and berberrubine for Alleviating heavy Metal-Induced male infertility in rats (111)	rats	5 mg/kg	90	F^3^	0.806mg/kg/d
14	Protective Effects of Annona Atemoya Extracts on Inflammation, Oxidative Stress, and Renal Function in Cadmium-Induced Nephrotoxicity in Wistar Rats (112)	rats	2 mg/kg	25	GH	0.323mg/kg/d
15	Attenuation of inflammation, oxidative stress and TGF-β1/Smad3 signaling and upregulation of Nrf2/HO-1 signaling mediate the protective effect of diallyl disulfide against cadmium nephrotoxicit (113)	rats	1.2 mg/kg	14	HJ	0.194mg/kg/d
16	Linalool may have a therapeutic effect on cadmium-induced nephrotoxicity by regulating NF-κB/TNF and GRP78/CHOP signaling pathways (114)	Rats	3 mg/kg	7	HJ	0.484mg/kg/d
17	Icariin reduces cadmium-induced renal injury in rats (115)	rats	1.5 mg/kg	10	H	0.242mg/kg/d
18	Curcumin protects against cadmium-induced germ cell death in the testis of rats (116)	rats	25 mg/kg	7	FH	4.032mg/kg/d
19	Alleviating Effects of Clove Essential Oil Disolved in Dimethyl Sulfoxide (Dmso) Against Cadmium-Induced Testicular and Epididymal Damages in Male Wistar Rats (117)	rats	20mg/kg	21	ABC^4^	3.226mg/kg/d
20	UNC93B1 Mediates the Effects of cGAS-STING on ER Stress and Iron Death, and Reduces Renal Toxicity in Cadmium-Exposed Rats (118)	rats	5 mg/kg	28	GH	0.806mg/kg/d
21	Therapeutic potential of the linalool against cadmium-induced testicular tissue damage (119)	rats	3 mg/kg	7	FI	0.484mg/kg/d
22	Effect of Arthrospira (Spirulina) maxima on Cadmium-Chloride-Induced Alterations in Sexual Behavior and Fertility in Male Wistar Rats (120)	rats	5 mg/kg	36	ABCH	0.806mg/kg/d
23	Anthocyanins from Lycium ruthenicum Murray Mitigate Cadmium-Induced Oxidative Stress and Testicular Toxicity by Activating the Keap1/Nrf2 Signaling Pathway (121)	mice	5 mg/kg	28	GH	0.407mg/kg/d
24	Renoprotective effect of hyperin against CdCl2 prompted renal damage by activation of Nrf-2/KeaP-1 ARE pathway in male mice (122)	mice	0.3 mg/kg	28	JH	0.024mg/kg/d
25	Low dose cadmium inhibits syndecan-4 expression in glycocalyx of glomerular endothelial cells (123)	mice	2.28 mg/kg	14	J	0.185mg/kg/d
26	Protective effect of quercetin on cadmium-induced kidney apoptosis in rats based on PERK signaling pathway (124)	rats	2 mg/kg	10	ABCDFHJ^5^	0.323mg/kg/d
27	Protective effect of quercetin on cadmium-induced kidney apoptosis in rats based on PERK signaling pathway (124)	rats	2 mg/kg	28	ABHJ	0.323mg/kg/d
28	Roflumilast extenuates inflammation and oxidative stress in cadmium-induced hepatic and testicular injury in rats (125)	rats	3 mg/Kg	5	ABH	0.484mg/kg/d
29	Benefits of Chlorella vulgaris against Cadmium Chloride-Induced Hepatic and Renal Toxicities via Restoring the Cellular Redox Homeostasis and Modulating Nrf2 and NF-KB Pathways in Male Rats (126)	rats	2 mg/kg	10	ABCGJ	0.323mg/kg/d
30	Toxicology of arsenate, arsenite, cadmium, lead, chromium, and nickel in testes of adult Swiss mice after chronic exposure by intraperitoneal route (127)	mice	1.5 mg/ kg	42	EFI	0.122mg/kg/d
31	Pomegranate peel extract, N-Acetylcysteine and their combination with Ornipural alleviate Cadmium-induced toxicity in rats (128)	rats	5 mg/kg/day		ABG	0.806mg/kg/d
32	[Effects of cadmium chloride on testicular autophagy and blood-testis barrier integrity in prepubertal male rats] (61)	rats	1 mg/kg	24h	ABGJ	0.161mg/kg/d
33	Morin mitigates cadmium-induced testicular impairment by stimulating testosterone secretion and germ cell proliferation in mice (68)	mice	10 mg/kg	35	ABCDEFGH^6^	0.813mg/kg/d
34	Atorvastatin prevents cadmium-induced renal toxicity in a rat model (129)	rats	1mg/kg	8	HJ	0.161mg/kg/d
35	Synthesized gold nanoparticles mediated by Crassocephalum rubens extract down-regulate KIM-1/NGAL genes and inhibit oxidative stress in cadmium-induced kidney damage in rats (130)	rats	20 mg/kg	5	HJ	3.226mg/kg/d
36	Lactoferrin alleviates spermatogenesis dysfunction caused by bisphenol A and cadmium via ameliorating disordered autophagy, apoptosis and oxidative stress (131)	mice	1.6 mg/kg		ABCDFHI	0.130mg/kg/d
37	[Effects of vitamin C on antioxidant function of testis in cadmium-loaded mice] (132)	mice	3 mg/kg	3	HI	0.244mg/kg/d
38	Renoprotective and Oxidative Stress-Modulating Effects of Taxifolin against Cadmium-Induced Nephrotoxicity in Mice (133)	mice	4 mg/kg	14	HIJ	0.325mg/kg/d
39	Comparative Potential of Zinc Sulfate, L-Carnitine, Lycopene, and Coenzyme Q10 on Cadmium-Induced Male Infertility (30)	rats	0.4mg/kg/day	3	ABDHIJ^7^	0.065mg/kg/d
40	Letrozole protects against cadmium-induced inhibition of spermatogenesis via LHCGR and Hsd3b6 to activate testosterone synthesis in mice (134)	mice	4 mg/kg	30	ABCDGI	0.325mg/kg/d

^1^ Effects: A, Decreased sperm motility; B, Reduced sperm density; C, Increased sperm abnormality rate; D, Sperm DNA damage; E, Disruption of the blood-testis barrier; F, Testicular damage; G, Alterations in sex hormone levels; H, Elevated oxidative stress levels; I, Apoptosis of spermatogenic cells; J, Renal function impairment. HED = Human Equivalent Dose. Tested agent: Cadmium chloride solution.

^2^ Effects: A, Decreased sperm motility; B, Reduced sperm density; C, Increased sperm abnormality rate; D, Sperm DNA damage; E, Disruption of the blood-testis barrier; F, Testicular damage; G, Alterations in sex hormone levels; H, Elevated oxidative stress levels; I, Apoptosis of spermatogenic cells; J, Renal function impairment. HED = Human Equivalent Dose. Tested agent: Cadmium chloride solution.

^3^ Effects: A, Decreased sperm motility; B, Reduced sperm density; C, Increased sperm abnormality rate; D, Sperm DNA damage; E, Disruption of the blood-testis barrier; F, Testicular damage; G, Alterations in sex hormone levels; H, Elevated oxidative stress levels; I, Apoptosis of spermatogenic cells; J, Renal function impairment. HED = Human Equivalent Dose. Tested agent: Cadmium chloride solution.

^4^ Effects: A, Decreased sperm motility; B, Reduced sperm density; C, Increased sperm abnormality rate; D, Sperm DNA damage; E, Disruption of the blood-testis barrier; F, Testicular damage; G, Alterations in sex hormone levels; H, Elevated oxidative stress levels; I, Apoptosis of spermatogenic cells; J, Renal function impairment. HED = Human Equivalent Dose. Tested agent: Cadmium chloride solution.

^5^ Effects: A, Decreased sperm motility; B, Reduced sperm density; C, Increased sperm abnormality rate; D, Sperm DNA damage; E, Disruption of the blood-testis barrier; F, Testicular damage; G, Alterations in sex hormone levels; H, Elevated oxidative stress levels; I, Apoptosis of spermatogenic cells; J, Renal function impairment. HED = Human Equivalent Dose. Tested agent: Cadmium chloride solution.

^6^ Effects: A, Decreased sperm motility; B, Reduced sperm density; C, Increased sperm abnormality rate; D, Sperm DNA damage; E, Disruption of the blood-testis barrier; F, Testicular damage; G, Alterations in sex hormone levels; H, Elevated oxidative stress levels; I, Apoptosis of spermatogenic cells; J, Renal function impairment. HED = Human Equivalent Dose. Tested agent: Cadmium chloride solution.

^7^ Effects: A, Decreased sperm motility; B, Reduced sperm density; C, Increased sperm abnormality rate; D, Sperm DNA damage; E, Disruption of the blood-testis barrier; F, Testicular damage; G, Alterations in sex hormone levels; H, Elevated oxidative stress levels; I, Apoptosis of spermatogenic cells; J, Renal function impairment. HED = Human Equivalent Dose. Tested agent: Cadmium chloride solution.

All 40 studies in **Table 1** were selected according to predefined inclusion/exclusion criteria (see Section 2). The human equivalent dose (HED) was calculated to extrapolate the minimal effective dose from animal studies to humans using the FDA−recommended body surface area normalization method (97). Studies such as Ref 104 were included because they met all inclusion criteria (*in vivo* design, oral cadmium exposure, ≥7 days duration, and clear reproductive/renal dose–response data).The No-Observed-Adverse-Effect Level (LOAEL) was converted to HED using the following formula: $HED(mg/kg)=Animal\;LOAEL\;(mg/kg)\times \frac{km\;of\;animal\;}{km\;of\;human}$The commonly used standard is: rat dose (mg/kg) ÷ 6.2=HED (mg/kg); mouse dose (mg/kg) ÷ 12.3=HED (mg/kg)(Refer to industry guidelines such as FDA (97)).

(The km factors of 6 and 37 for rat and human (60 kg), respectively, were applied as per standard recommendations.According to **Table 1**, the smallest HED was calculated to be 0.002 mg/kg/d.) (135).

## 3 Sources and pathways of cadmium exposure

### 3.1 Environmental media exposure pathways

#### 3.1.1 Inhalation via respiratory tract

Industrial pollution and tobacco smoke are the main ways cadmium is inhaled by air. Industrial pollution sources constitute a major pathway for human cadmium (Cd) inhalation. Industrial activities such as mining, metallurgy, and battery manufacturing release cadmium-containing aerosols, which are directly inhaled through the respiratory tract. Studies have shown that urinary cadmium levels in some industrial-intensive areas are significantly higher than the national average (machine models predict that the peak urinary cadmium level in industrial counties in 2024 will be 1.09 μg/g Cr). Tobacco smoke represents another significant source of airborne Cd exposure. Each cigarette contains 0.5–1 μg of Cd, resulting in blood cadmium concentrations in smokers being 2–3 times higher than in non-smokers (14). A study using the risk assessment model of the United States Environmental Protection Agency (USEPA) found that the global average daily dietary Cd intake was 2.20 μg/kg bw/d (15). The daily exposure to cigarette Cd was 0.0359 μg/kg bw/d. Cadmium is rapidly absorbed into the bloodstream via the alveoli, exacerbating systemic accumulation. It can be seen that industrial pollution and tobacco smoke are important ways for cadmium to be inhaled by air.

#### 3.1.2 Transfer through the soil-crop system

The soil-crop system is also an important route for human ingestion of cadmium. Crops such as rice, wheat, and leafy vegetables (e.g., lettuce, peppers) cultivated in Cd-contaminated soil exhibit high bioaccumulation factors. Soil cadmium pollution can lead to Cd levels in agricultural products exceeding food safety limits, compromising product quality and safety. The 2023 evaluation of wheat samples in one region showed that the cadmium exceeded the standard rate of 1.89%. Although staple foods are classified as mildly contaminated (composite pollution index of 1.737), they account for 57.51% of the total dietary Cd intake (16). Soil cadmium accumulation mainly derives from mining, smelting, and irrigation with contaminated water. Under the prevalent double-cropping rice system in southern China, irrigation represents a major source of cadmium (Cd) input, contributing approximately 1 to 400g Cd ha^-1^ yr^-1^ (17).Additional Cd inputs arise from atmospheric deposition (0.4–25 g Cd ha^-1^ yr^-1^), application of organic fertilizers (0–10 g Cd ha^-1^ yr^-1^), and phosphorus-based fertilizers (0.04–2 g Cd ha^-1^ yr^-1^). In contrast, the annual removal of Cd through the harvest of two rice crops is estimated at only 5 to 55g Cd ha^-1^ yr^-1^. Furthermore, Cd bioavailability in soil exhibits distinct regional patterns. It is generally higher in acidic soils typical of southern provinces such as Hunan and Jiangxi, leading to average Cd concentrations in rice grains that are approximately 40% greater than those observed in northern regions (11).

To better illustrate the Cd balance, the major input and output fluxes are summarized in **Table 2**.

**Table 2:** Annual cadmium fluxes (g ha^-1^ yr^-1^) in double-cropping rice systems of southern China.

Flux type	Source	Range
Input	Irrigation	1–400
	Atmospheric deposition	0.4–25
	Organic fertilizers	0–10
	Phosphorus fertilizers	0.04–2
Output	Rice grain harvest	5-55

### 3.2 Dietary exposure

Due to its high soil-to-plant transfer efficiency, cadmium is widely present in plant-derived foods (18–20). For non-smoking individuals without occupational exposure, diet is the predominant exposure pathway, contributing up to 90% of the total body cadmium burden (21). Heavy metals can also accumulate in livestock products such as meat and milk, forming another important exposure route for humans.

Among various global diets, rice represents the largest source of dietary Cd intake, although the level varies significantly by region and country. For instance, in Europe, rice contributes to less than 27% of the total dietary Cd intake for the general population (22), compared to approximately 44% in Japan (23) and 56% in China. This substantial contribution of rice to total dietary intake arises from its high proportional consumption (especially in Asian countries) combined with its relatively high Cd concentration compared to other cereal-based foods (24).

International Safety Thresholds and Exposure Limits (JECFA Standard):The Provisional Tolerable Monthly Intake (PTMI) is established at 25 μg/kg BW/month, corresponding to a urinary cadmium threshold of 5–10 μg/g creatinine (25). Actual Exposure Level: The estimated monthly dietary cadmium intake among middle-aged and elderly populations in China averages 7.39 μg/kg BW/month, remaining below the PTMI. Although the average dietary intake is lower than PTMI, dietary intake is only one of the ways of cadmium intake, and the average may mask the local high risk level. In high-exposure areas or specific populations, such as residents in areas where high-cadmium rice is eaten, the intake of cadmium may be close to or exceed PTMI. Nevertheless, vigilance against composite toxicity is warranted in areas with co-contamination of chromium, such as Northern Anhui Province (26). This concern is supported by growing evidence that co-exposure to cadmium and chromium can produce synergistic effects, particularly by exacerbating oxidative stress and organ damage, thereby posing a greater health risk than exposure to either metal alone (27, 28).

## 4 Cadmium-induced damage to male reproductive function and its mechanisms

### 4.1 Semen parameters

Research has found that exposure to cadmium (Cd) and/or other environmental toxicants can negatively impact the fertility of male animals, manifesting as reduced sperm count and poor semen quality (29). Cadmium exposure decreases sperm count, progressive sperm motility, testosterone concentration, and the activities of superoxide dismutase (SOD) and catalase, as well as glutathione (GSH) content. It also induces vacuolization of the seminiferous tubules and oligospermia in rat testes (30). Therefore, elevated levels of cadmium in semen may be a contributing factor to male infertility in exposed populations. Chronic cadmium exposure via drinking water in sexually mature rats demonstrated a dose-dependent decrease in sperm motility within the cauda epididymis and a concurrent increase in the percentage of immotile sperm. Doses ≥100 mg/L Cd significantly inhibited sperm motility, reduced sperm density in the cauda epididymis, and markedly increased the sperm abnormality rate (31). Morphological analysis reveals that sperm abnormalities can occur in the head, tail, or midpiece, with head defects being the most prevalent among abnormal spermatozoa.

### 4.2 Increased sperm DNA fragmentation

The integrity of sperm DNA is a critical factor for successful fertilization and fertility. Sperm DNA can be damaged by endogenous and exogenous factors, jeopardizing the development of a healthy fetus (32). Studies (33) both domestically and internationally demonstrate a significant correlation between sperm DNA integrity and sperm function. Experimental research investigating the effects of cadmium on epididymal sperm parameters and sperm DNA in mice found that different doses of Cd caused varying degrees of damage to testicular histomorphology at different time points post-exposure. Cadmium chloride administration for 10 weeks was shown to damage epididymal sperm DNA, with the severity of damage increasing in a dose-dependent manner.

## 5 Potential mechanisms of cadmium reproductive toxicity

Cadmium-induced male reproductive toxicity is driven by a unified, sequential cascade of interconnected pathways rather than isolated events. First, cadmium enters testicular cells through calcium and divalent metal ion channels, triggering ionic imbalance and oxidative stress. Excessive oxidative stress further induces endoplasmic reticulum stress, which regulates autophagy and apoptosis in spermatogenic cells. Together, these processes disrupt structural proteins of the blood-testis barrier, destroying the microenvironment required for normal spermatogenesis. Meanwhile, cadmium-induced renal injury reduces cadmium excretion and increases systemic cadmium accumulation, creating a vicious cycle that amplifies testicular damage. All pathways converge to impair sperm production, quality, and DNA integrity, ultimately leading to male reproductive dysfunction.

### 5.1 Cellular Level

As the initiating step in the unified mechanistic cascade, at the cellular level, cadmium induces damage and repair processes in which cellular redox status plays a critical role. It is clearly established that Cd can cause indirect oxidative damage to DNA (34). Gene mutations triggered by Cd exposure primarily affect genes associated with cell proliferation, apoptosis, and DNA repair (35). Its toxic effects are mainly exerted by influencing DNA repair genes and proliferation-related genes; mutations and dysregulation in these genes may lead to malignant transformation of cells (36). Previous studies have found that Cd exposure can alter the methylation levels of specific loci or genes: hypomethylation of MGMT is associated with increased Cd levels in humans and exhibits sex differences (37); low-dose and high-dose Cd exposure decrease and increase the methylation level of the KLOTHO gene, respectively (38); hypermethylation of *RASAL1* is positively correlated with Cd exposure (39). Furthermore, studies (40) utilizing epigenome-wide association analysis (EWAS) have analyzed the association between urinary cadmium and DNA methylation, identifying differentially methylated positions (DMPs) associated with urinary Cd. Concurrently, they analyzed the relationship between urinary Cd-associated DMPs and kidney injury biomarkers, identifying overlapping DMPs. Building on this, further exploration revealed the mediating role of DNA methylation in the association between urinary Cd and kidney injury indicators, uncovering the pathway “Cd exposure — DNA methylation — Kidney injury,” thereby identifying potential early intervention targets for Cd exposure and kidney injury. Oxidative stress is a conserved mechanism of heavy metal toxicity, and natural products such as propolis can alleviate heavy metal-induced damage by enhancing antioxidant capacity (41–43).

### 5.2 Testicular injury

As a central downstream event in the integrated reproductive toxicity pathway, cadmium can directly damage the testes in rodent models. The extent of the decrease in daily sperm production (DSP) worsens with prolonged Cd exposure (44). Cd impairs testicular development and spermatogenesis and can enhance endoplasmic reticulum stress (ERS) within the testes, leading to germ cell damage. Research results indicate that Cd can increase testicular ERS and autophagy levels. Moreover, Cd may primarily induce testicular ERS via the PERK-eIF2α-ATF4-CHOP pathway (45). Using GC-1 cells as a model, this study further investigated the effect of Cd on spermatogonial cell proliferation and the cell cycle. Experimental results demonstrated that Cd exposure caused S-phase arrest in the GC-1 cell cycle and inhibited GC-1 cell proliferation, with the inhibitory effect strengthening as Cd concentration increased.

### 5.3 Induction of cellular autophagy

Within the integrated toxicity cascade, a complex and critical link exists between endoplasmic reticulum stress (ERS) and autophagy (46). Low-dose Cd exposure may induce autophagy as a cytoprotective mechanism, whereas prolonged Cd exposure may disrupt the autophagic process and lead to apoptosis (47). Cd activates the ERS PERK-eIF2α-ATF4-CHOP pathway, and ERS can mediate autophagy activation (48). Additionally, Cd can increase the expression of ERS regulators CHOP, eIf2α, and ATF4, activating autophagy and upregulating LC3 expression levels (49). ERS can mediate Cd-induced spermatogonial cell apoptosis (50). This study further explored the role of ERS-induced autophagy in Cd-mediated inhibition of GC-1 cell proliferation. Experiments found that inhibiting either ERS or autophagy in GC-1 cells significantly alleviated the Cd-induced reduction in cell cycle-related protein expression levels and mitigated the inhibitory effect of Cd on cell proliferation. Research results indicate that Cd can inhibit spermatogonial cell proliferation via the ERS-induced autophagy pathway (45).

### 5.4 Disruption of the blood-testis barrier

As a key structural target in the unified mechanistic model, the blood-testis barrier (BTB) provides a specialized microenvironment for germ cells that is indispensable for normal spermatogenesis (51). The mammalian BTB is primarily constituted by tight junctions, adherens junctions, and gap junctions between adjacent Sertoli cells near the basement membrane of the seminiferous tubules (52). Each junction type performs irreplaceable physiological functions. Tight junctions confer cell polarity and form a watertight barrier preventing paracellular leakage of water, ions, and other molecules (53). Adherens junctions form a continuous adhesion belt beneath the tight junctions, linking neighboring cells, except where they coexist with tight junctions (54). Gap junctions regulate spermatogenesis and facilitate intercellular communication (55). The BTB plays a critical role in spermatogenesis and its maintenance. Tight junction proteins are thought to form oligomers through homotypic and heterotypic interactions of tight junction proteins on adjacent Sertoli cells, constituting the tight junction strands (56).

In occludin(−/−) mice, tight junction strands fail to assemble, and mice with dysfunctional BTB are infertile (57). Spermatogenesis in claudin-11(−/−) mice cannot progress beyond the meiotic stage (58). Connexin43 (Cx43) plays a crucial role in germ cell development; its deletion leads to reduced fetal germ cell numbers and inhibits spermatogenesis in adult Sertoli cells (59). Studies suggest an association between increased cell adhesion/gap junction protein expression and BMP4 (bone morphogenetic protein 4)-induced spermatogonial stem cell (SSC) differentiation (60), supporting the hypothesis that an intact BTB provides and maintains the appropriate microenvironment for spermatogonial differentiation. One study found that cadmium chloride induced autophagy in the testes of male rats and disrupted BTB integrity (61). Furthermore, Cd accumulates in mice and inhibits testosterone; Cd concentrations increased significantly proportionally to exposure levels in serum and testes. Compared to low-dose Cd-treated mice, Cd exposure significantly reduced serum testosterone levels in high-dose Cd-treated mice (62).

### 5.5 Ionic damage

As the primary upstream gateway in the integrated toxicity cascade, evidence indicates that cadmium enters testicular cells through various ion channels and transporters, including channels for calcium, iron, and zinc (63). Research shows that multiple calcium (Ca^2+^) and potassium (K⁺) channel subtypes exist in the human testis and sperm and are involved in the early stages of the acrosome reaction (64). Notably, Ca^2+^ channels are susceptible to the toxic effects of cadmium ions (Cd^2+^), while K⁺ channels are susceptible to lead ions (Pb^2+^) (65). These ion channels provide potential entry routes for metallic toxicants into mature sperm (66). Polymorphisms in ion channels may contribute to individual differences in susceptibility to Cd^2+^ and Pb^2+^ toxicity (65). Patients with varicocele-associated infertility exhibit higher Cd^2+^ levels, while those with unexplained infertility exhibit higher Pb^2+^ levels. Notably, both types of male infertility patients showed a reduced capacity for the acrosome reaction. Measuring specific Ca^2+^ and K⁺ channel subtypes via reverse transcriptase-polymerase chain reaction (RT-PCR) might help identify susceptible individuals with lower resistance to environmental exposures like Cd and Pb (12).

**Figure 1:**
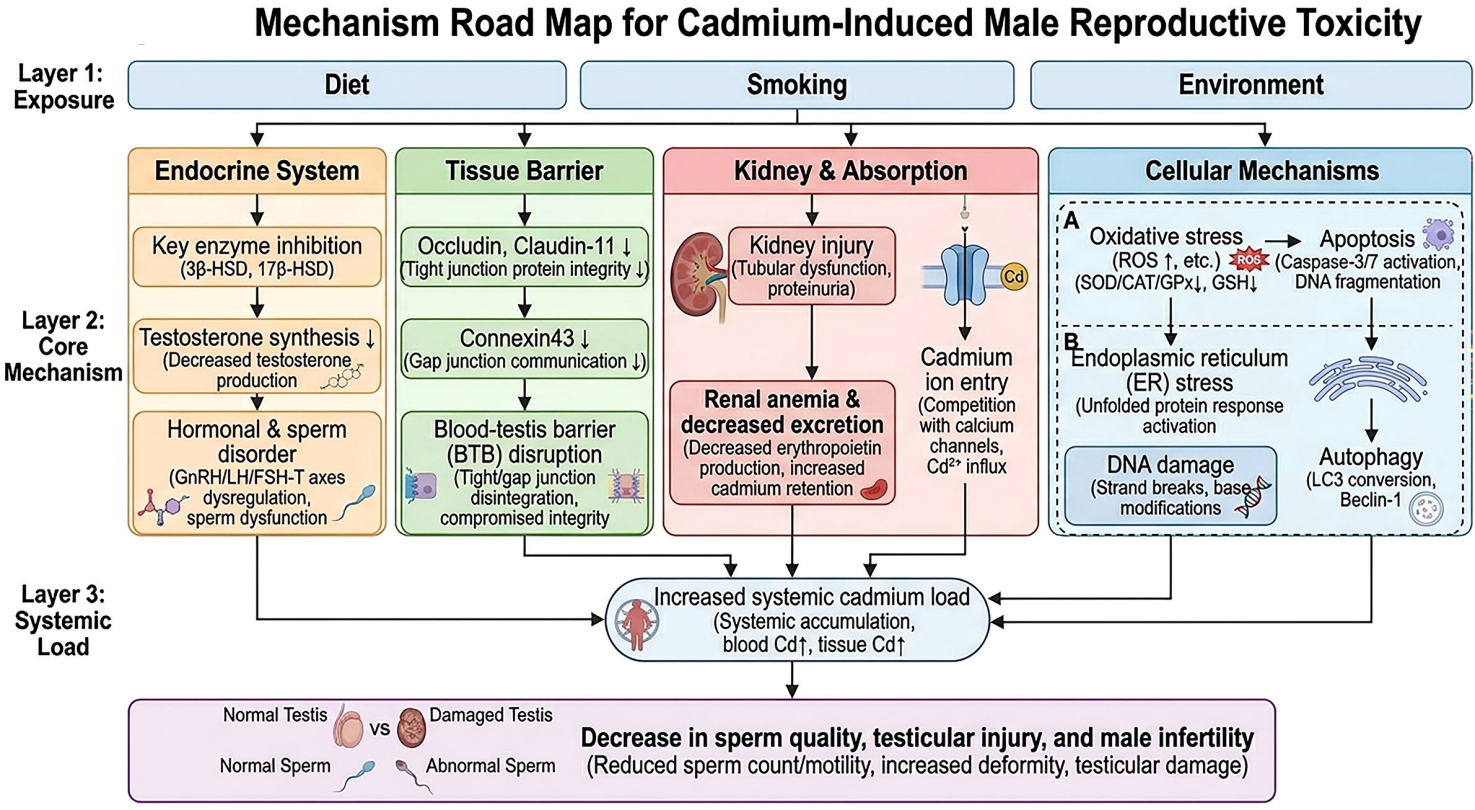
Mechanism of cadmium-induced male reproductive toxicity.

Additionally, regarding the molecular mechanisms of testicular injury induced by environmental toxicants like Cd (and Bisphenol A), research has revealed that the initial point of action is the disruption of the blood-testis barrier (BTB). This disruption subsequently impairs germ cell adhesion, ultimately leading to germ cell loss, reduced sperm count, and male infertility (67). Study results indicate testicular disorders, decreased circulating testosterone levels, reduced sperm density, and elevated oxidative stress and sperm abnormalities in Cd-intoxicated mice. The expression of germ cell proliferation markers, germ cell nuclear acidic protein (GCNA), and the adipocytokine visfatin was also downregulated (68). **Figure 1**.

## 6 Systemic effects and combined exposure

### 6.1 Renal function impairment

Previous studies have found that the mechanism by which chronic cadmium exposure indirectly induces male reproductive toxicity through renal injury can be summarized as two complementary pathways: on the one hand, cadmium selectively damages the proximal renal tubules, inhibits erythropoietin (EPO) synthesis, and disrupts the VitD calcium phosphate axis, causing renal anemia and tissue hypoxia, thereby weakening the hypothalamic pituitary testicular axis pulsatile GnRH/LH/FSH secretion, downregulating StAR, P450sc, and 3 β - HSD expression, leading to testicular interstitial cell dysfunction and significant decrease in serum testosterone, resulting in renal hypogonadism (69);On the other hand, the kidneys are both the main reservoir and excretion channel of cadmium. When the cadmium load in the renal cortex exceeds 200 mg/kg or eGFR decreases, renal excretion function declines, and the half-life of blood cadmium is extended from about 20 years to more than 30 years. The systemic circulating cadmium load increases, exposing distant reproductive organs such as the testes to secondary exposure, exacerbating oxidative stress and mitochondrial damage, forming a vicious cycle of “kidney damage - elevated blood cadmium - testicular re attack” (70–72) These two pathways synergistically amplify the direct reproductive toxicity caused by cadmium. This renal−testis axis represents a critical endocrine−toxicological mechanism that exacerbates dysfunction of the hypothalamic−pituitary−testicular (HPT) axis and increases overall reproductive risk.

### 6.2 The detrimental effects of combined cadmium and microplastic exposure on male reproduction

Human exposure to environmental contaminants in daily life is rarely singular; male reproductive system damage is often the result of combined effects from multiple pollutants. Microplastics (MPs) and lead (Pb) are also common environmental contaminants contributing to male reproductive impairment. Co-exposure to cadmium (Cd) with MPs and Pb can further exacerbate damage to the male reproductive system.

Microplastics in real environmental matrices frequently adsorb toxic substances such as heavy metals. This co-exposure may induce diverse negative impacts on human reproduction (73). A systematic review indicates a substantial global decline in sperm count (approximately 62% between 1973 and 2018), which is associated with increased exposure to environmental toxicants. Sperm concentration fell from 101 million per milliliter (mL) to 49 million/mL, representing a 51.6% reduction. Notably, since 2000, the average annual decline rate has accelerated to 2.64% (74). This phenomenon is closely associated with increased exposure to environmental toxicants, including heavy metals, endocrine-disrupting chemicals, and MPs (75). Plastic waste can fragment into microplastics (MPs, ≤ 5mm in diameter or length) and nanoplastics (NPs, ≤ 100 nm in diameter or length) through natural physicochemical processes or biodegradation. MPs have been detected in diverse environments, encompassing marine ecosystems, freshwater systems, the atmosphere, food, and drinking water (76). Most studies suggest an inverse relationship between MP size and toxicity (77). Physicochemical studies confirm that MPs can adsorb the heavy metal cadmium (Cd) (78). Co-exposure to MPs and Cd induces more severe oxidative damage and cellular apoptosis compared to the effects of either substance alone (79). Research demonstrates that 0.1 μm polystyrene (PS) exhibits stronger toxicity than 1 μm PS, while the combined toxicity of 0.1 μm and 1 μm PS exceeds that of individual exposures. Combined exposure to MPs and CdCl_2_ results in more pronounced toxic effects on male reproductive function (80).

Co-exposure to PS-NPs and Cd may impair spermatogonia by reducing cell viability, inducing morphological abnormalities, and triggering ferroptosis, potentially through disruption of the HIF-1α-associated signaling axis (miR-199a-5p–HIF-1α–iron metabolism) in GC-1 cells (73). Iron is crucial for normal mammalian spermatogonia during rapid spermatogenic cell division (81). However, iron overload impairs testicular development and spermatogenesis in mice and reduces spermatogonial cell survival (82). Simultaneously, spermatogenesis demands substantial oxygen and energy; insufficient oxygen levels can lead to metabolic damage in the reproductive system. Hypoxia-inducible factor 1α (HIF-1α) is a transcription factor highly sensitive to hypoxic conditions, directly involved in regulating tissue and cellular responses to hypoxia (75). Studies indicate that di-(2-ethylhexyl) phthalate (DEHP) induces ferroptosis in mouse testes via the HIF-1α/heme oxygenase-1 (HO-1) signaling pathway (83). Notably, such synergistic toxicity between cadmium and microplastics can lower the actual reproductive toxic threshold, suggesting that the HED calculated from single-cadmium exposure may underestimate the real health risk under real-world mixed exposure conditions.

### 6.3 The cooperative toxicity of cadmium and lead on male reproductive damage

Cadmium (Cd) and lead (Pb), as widely coexisting heavy metal pollutants in the environment, exacerbate male reproductive damage through synergistic interactions. The core mechanisms involve suppression of hormone synthesis, induction of oxidative stress, dysregulation of metallothionein (MT), and induction of prostatic lesions.

#### 6.3.1 Testicular steroidogenesis impairment and sperm parameter deterioration

Combined Pb and Cd exposure disrupts testicular function through a dual mechanism: inhibition of steroidogenesis and impairment of spermatogenesis. In a subchronic exposure model (Pb(NO_3_)_2_ + CdCl_2_, 269.65 mg/kg, 90 days), Pb^2+^ and Cd^2+^ competitively inhibit Ca^2+^ channels, interfering with calcium-dependent signaling (84). This leads to reduced activity of key testosterone-synthesizing enzymes: 3β-HSD activity decreased and 17β-HSD activity decreased by 49% (P<0.01). Consequently, serum testosterone declined by 41% (P<0.01) and intratesticular testosterone decreased by 38% (P<0.01) (85). Spermatogenesis was concurrently impaired: combined exposure (Pb + Cd, each 0.025 mg/kg/day, intraperitoneal injection, 15 days) caused a sharp 27% reduction in testicular sperm count (119.6×10^6^ → 85.4×10^6^, P<0.01), a 32% decrease in epididymal sperm motility (P<0.01), and a 1.8-fold increase in sperm abnormality rate. Histological examination revealed extensive vacuolization in seminiferous tubules and disarrangement of Sertoli cells, directly correlating with oxidative stress-induced apoptosis of germ cells (86). Human studies further corroborate this: smokers (tobacco contains 0.5–2 μg Cd/cigarette) exhibit significantly elevated seminal Cd burden (r^2^=0.07, P<0.05), and sperm motility shows a dose-dependent inverse correlation with Qmax values (r^2^=0.095, P<0.05) (87), highlighting the clinical association between environmental exposure and reproductive damage.

#### 6.3.2 Oxidative stress cascade and antioxidant defense collapse

Combined Pb and Cd exposure induces an oxidative stress cascade in testicular and prostatic tissues, leading to a comprehensive collapse of the antioxidant defense system. Many natural substances with phenolic components exhibit strong antioxidant activity against heavy metal toxicity.

##### 6.3.2.1 Exacerbated lipid peroxidation across organs

In testicular tissue, Cd exposure alone increased mitochondrial malondialdehyde (MDA) by 152%, while combined (Pb+Cd) exposure still elevated mitochondrial MDA by 51%. Mitochondrial damage was significantly more severe than cytoplasmic damage (MDA increase: mitochondria 51% vs. cytoplasm 29%), confirming mitochondria as the primary target of ROS (85). Prostatic tissue similarly exhibited a strong positive correlation between LPO and Cd content (mitochondria r^2^=0.128, P<0.01; cytoplasm r^2^=0.097, P<0.05). The underlying mechanism involves Cd^2+^/Pb^2+^ catalyzing the OH radical chain reaction in membrane polyunsaturated fatty acids (88).

##### 6.3.2.2 Imbalance in the antioxidant enzyme system

In testes, superoxide dismutase (SOD) activity decreased by 32% (mitochondrial) and 49% (cytoplasmic) after combined exposure, attributed to Pb^2+^ competitively displacing essential metal ions in the Cu/Zn-SOD active site (89). Catalase (CAT) activity decreased by 64% (85), leading to H_2_O_2_ accumulation and further inactivation of the enzyme protein (90). Conversely, glutathione peroxidase (GPx) activity increased paradoxically (161% in mitochondria, 262% in cytoplasm), reflecting a compensatory stress response to clear H_2_O_2_ (91). In prostatic tissue, GPx activity also significantly increased with Cd accumulation (mitochondrial r^2^=0.177, P<0.01) (87), highlighting the conserved nature of the response pattern across organs.

##### 6.3.2.3 Depletion of non-enzymatic antioxidants

Testicular mitochondrial glutathione (GSH) decreased by 22-28%, and cytoplasmic GSH decreased by 28-35% (85). This occurs because Cd/Pb binds to the -SH groups of GSH, forming metallothiolate complexes (92). Hepatic and renal GSH also significantly decreased (70% in liver, 58% in kidney of Cd-exposed groups) (93), confirming the systemic spread of oxidative stress.

##### 6.3.2.4 Clinical association with benign prostatic hyperplasia

Prostate-related disorders have been found to have a significant impact on male reproductive health, with effects on testicular volume, testosterone levels, testosterone levels, semen quality, and sexual performance (94). Epidemiological studies reveal that the specific accumulation of cadmium (Cd) in prostatic tissue exhibits a significant positive correlation with the severity of benign prostatic hyperplasia (BPH). Data from a Western Indian cohort (n=116) show that for every 0.1 μg/g increase in prostatic Cd content, the maximum urinary flow rate (Qmax) decreases by 23% (r^2^=0.095, P<0.05). Histopathology further confirmed that the high-Cd group (>0.13 μg/g) had a 40% increase in the number of acini and exacerbated epithelial hyperplasia. In contrast, lead (Pb), despite higher accumulation levels (mean 12.5 μg/g), showed no significant correlation (r^2^=0.003, ns) (87). Smoking acts as a key synergistic factor: tobacco smoke (containing 0.5–2 μg Cd/cigarette) increases prostatic Cd burden in smokers by 68% compared to non-smokers (0.077 vs. 0.024 μg/g, P<0.05) and leads to a 21% reduction in Qmax (P<0.05). The molecular mechanism involves Cd^2+^ mimicking estrogen by binding to the estrogen receptor (ERα) (95) while simultaneously activating the androgen receptor (AR) signaling pathway (96), synergizing with endogenous testosterone to promote proliferation of prostatic stromal and epithelial cells (87). Notably, while Pb does not directly participate in BPH progression, it indirectly amplifies Cd toxicity by inducing oxidative stress (e.g., 70% increase in mitochondrial lipid peroxidation) (85). Moreover, occupationally exposed populations (e.g. battery factory workers) concurrently exposed to Pb/Cd aerosols face a 2.3-fold increased risk of prostatic lesions (12). This synergistic amplification of reproductive toxicity further demonstrates that the HED based on single cadmium exposure represents a conservative estimate, and actual human health risks may be substantially higher due to combined exposure to multiple toxicants.

## 7 The association between current human cadmium exposure levels and damage to the male reproductive system

Currently, the dose–response relationship for cadmium-induced male reproductive toxicity remains incompletely defined. Forty eligible *in vivo* studies were systematically selected based on predefined criteria (see Section 2). Based on the lowest observable adverse effect level (LOAEL, 10 μg/kg/d) from a 90−day oral rat study (Ait Benbella et al., 2025), the human equivalent dose (HED) was calculated using the body surface area normalization method. The human equivalent dose (HED) was calculated to extrapolate the minimal effective dose from animal studies to a potential dose in humans. The calculation was based on the body surface area normalization method as recommended by the U.S. Food and Drug Administration (FDA) guidance (97). We compiled data from studies reporting detrimental effects of Cd on the male reproductive system in rodents and calculated the corresponding Human Equivalent Dose (HED) for cadmium exposure based on body surface area. However, this calculation method still has potential limitations and uncertainties in extrapolating cadmium reproductive toxicity across species. For example, there may be differences in cadmium metabolism, detoxification, and target organ sensitivity among different species. Further animal experiments and more in-depth research are needed to verify this. Details are provided in **Table 1**.

**Figure 2:**
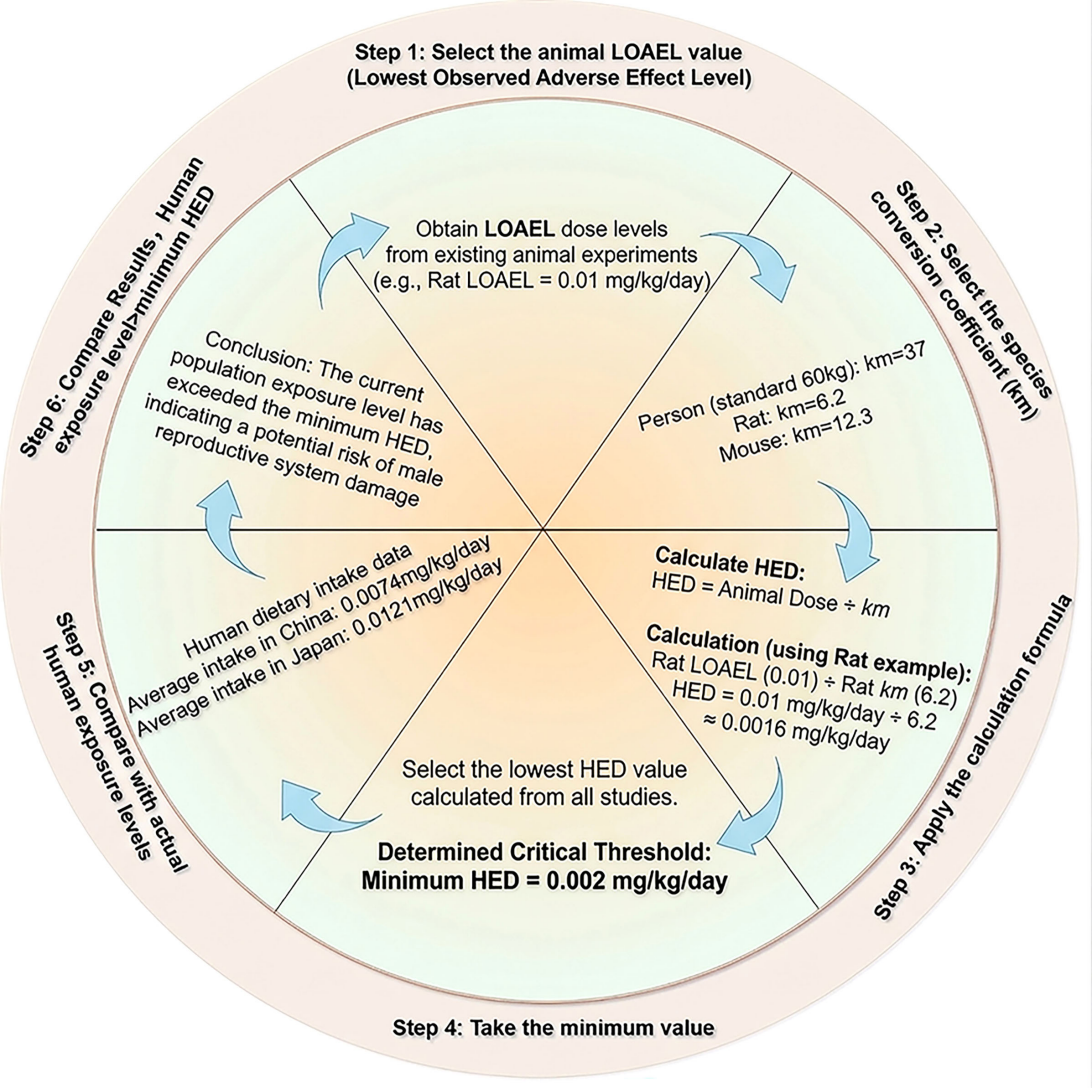
Calculation process and principle flowchart of human minimum equivalent dose (HED).

According to the predefined selection criteria for chronic reproductive toxicity (sexually mature rodents, ≥90 days of oral exposure, and comprehensive reproductive endpoints), Study 1 in **Table 1** (Ait Benbella et al., 2025) was selected because it employed 90-day chronic exposure in adult rats and evaluated core reproductive indicators including sperm quality and blood-testis barrier integrity. The lowest dose causing statistically significant reproductive damage was 10 μg/kg/day, representing the lowest observable adverse effect level (LOAEL); this dose induced a 54% decrease in sperm motility, a 65% decrease in sperm density, sperm DNA damage, disruption of the blood-testicular barrier, and an 11% decrease in testicular weight in rats, and the data in this study were expressed as mean ± standard deviation, and all ANOVA tests showed a Shapiro-Wilk normal P-value > 0.05, all ANOVA tests yielded a Levene test P-value > 0.05, statistically analyzed using one-way analysis of variance (ANOVA). A P-value < 0.05 was considered statistically significant. P-This LOAEL (10 μg/kg/d) was used to calculate the critical minimum human equivalent dose (HED) using body surface area normalization, and the corresponding HED was 0.002 mg/kg/d = 2 μg/kg/day. Other studies in **Table 1** with numerically lower HED values were not chosen as the critical threshold because they either focused only on renal injury, lacked sperm or testicular toxicity endpoints, used acute high-dose exposure, or had an exposure duration shorter than 7 days, which cannot represent long-term chronic environmental cadmium exposure in humans. Recent studies on dietary cadmium exposure levels in China show that the average daily dietary cadmium intake reaches 7.39 μg/kg/day = 0.0074 mg/kg/day, which far exceeds the minimum dose of 0.002 mg/kg/day that causes a decline in sperm quality in rats (25). Similarly, the study of cadmium intake in Japanese adults showed a geometric mean (geometric standard deviation) of 12.1 μg/kg/day = 0.0121 mg/kg/day), also exceeding 0.002 mg/kg/day (98). According to the average intake of the above populations was 7.39 μg/kg/day and 12.1 μg/kg/day, respectively, it was significantly higher than the lowest HED (2 μg/kg/day), and 3.7 times and 6 times higher than the lowest HED (2 μg/kg/day), respectively, current exposure levels are significantly higher than the minimum threshold for observed reproductive damage. At the same time, it is also necessary to consider the significant variability in population exposure, and the average intake may underestimate the actual exposure level of certain populations such as residents of high-pollution areas, people with specific diets, smokers, etc., who are at higher risk of cadmium exposure. In addition to this, diet is only one of the routes of exposure, and other routes such as smoking, occupational exposure, and air inhalation further increase the total load. Therefore, cadmium exposure levels in some populations approach or exceed this minimum HED threshold, which suggests a potential risk of male reproductive system dysfunction. Furthermore, given the synergistic reproductive toxicity of cadmium combined with microplastics, lead, and other environmental pollutants and the amplifying effect of the renal−testis axis, the actual reproductive health risk may be higher than the current HED estimation based on single exposure. **Figure 2**.

## 8 Summary and future perspectives

Humans are exposed to cadmium through multiple routes. Chronic cadmium intake has been associated with adverse impacts on male reproductive capacity in animal studies and epidemiological observations, and exposure via dietary sources and cigarette smoking also may significantly contribute to the burden of cardiovascular diseases and cancer risk, posing substantial threats to human health. This study systematically reviewed recent relevant research to identify the primary mechanisms of cadmium-induced damage to male reproductive function, including the critical renal−testis axis by which kidney injury secondarily exacerbates testicular toxicity and disrupts the hypothalamic−pituitary−testicular axis. Using the body surface area normalization method, we calculated the corresponding minimum biologically effective level (human equivalent dose) for human exposure. Notably, current cadmium exposure levels in some countries are approaching this calculated minimum human equivalent dose, reflecting changes and developments in modern lifestyles. Furthermore, we outline future research directions: Our team will conduct further animal experiments to validate these findings and assess the impact of long-term, low-dose cadmium exposure on male reproductive capacity, based on current human exposure levels. We will also strive to elucidate the precise mechanisms underlying cadmium-induced reproductive damage to identify effective therapeutic strategies. Additionally, research will focus on interventions targeting cadmium exposure and intake pathways, aiming to develop methods for reducing and eliminating cadmium from the human body.

## Data Availability

The datasets presented in this study can be found in online repositories. The names of the repository/repositories and accession number(s) can be found in the article/supplementary material.
